# Using Existing Software Systems to Collect Outcome Data in Adult Day Services

**DOI:** 10.1093/geroni/igab046.321

**Published:** 2021-12-17

**Authors:** Keith Anderson, Lisa Peters-Beumer, Laurie Duff

**Affiliations:** 1 University of Texas at Arlington, Arlington, Texas, United States; 2 Concordia University Chicago, River Forest, Illinois, United States; 3 Easterseals New Hampshire, Manchester, New Hampshire, United States

## Abstract

Recently, there has been a resounding call for standardized outcome data collection in adult day services (ADS). Outcome data have the potential to demonstrate the effectiveness of ADS, aid in program development, and help leverage funding opportunities. Unfortunately, many ADS centers do not collect outcome data for several reasons, including the cost of data collection software and systems. In this presentation, we present one effort to utilize an existing multiuse ‘off the shelf’ software solution to collect ADS outcome data for a network of ADS providers. The researchers collaborated with software developers and ADS providers to adapt the software to incorporate outcome measures and reporting functionality on both the individual and program levels. Adaptation and adoption required attention to HIPAA compliance, workflow integration, measurement fidelity, and data management processes. Despite these challenges, adapting existing software systems may be a cost-effective way to enable expanded outcome data collection in ADS.

